# Relationships between freeze tolerance and plant architecture in winter wheat during tillering stage

**DOI:** 10.3389/fpls.2026.1745479

**Published:** 2026-02-17

**Authors:** Han Wang, Bai-Song Yang, Li-Wei Xing, Shu-Ying Yang, Shu-Nv Hao, Hui-Hui Zhang, Wan-Ke Yu, Xiao-Neng Wan, Kai-Di Lyu, Xin Ma, Jia Luo, Zhi-Yu Fang, Min Yang, Guo-Zhong Sun

**Affiliations:** 1 College of Agriculture and Biotechnology, Hebei Normal University of Science and Technology, Qinhuangdao, China; 2 National Engineering Research Center for Crop Molecular Breeding, Institute of Crop Sciences, Chinese Academy of Agricultural Sciences, Beijing, China; 3 Hailar District Agricultural and Forestry Technology Promotion Center, Hailar Agriculture and Animal Husbandry Bureau, Hulunbuir, China

**Keywords:** freeze tolerance, mortality rate of shoots, severity of leaf necrosis, tiller angle, *Triticum aestivum* L.

## Abstract

Winter freezing injury is a critical factor limiting wheat(*Triticum aestivum* L.) productivity in northern China. Since freeze tolerance (FT) correlates with seedling growth traits, this study investigated the relationship between FT and plant architecture (PA) in winter wheat at the tillering stage. We evaluated 550 wheat varieties and advanced lines from the Huang and Huai River Valleys Winter Wheat Zone of China. Seedling PA was classified using the International Union for the Protection of New Varieties of Plants (UPOV) standards, while FT was evaluated through two parameters: severity of leaf necrosis (SLN) and mortality rate of shoots (MRS). The results showed that the PA distribution across germplasms approximated a normal distribution. The relationships between SLN and MRS under freezing stress were highly variable across years with differing winter conditions. SLN and MRS-derived FT levels showed a positive correlation within the same growing season but were inconsistent across different years. PA and MRS showed no correlation whereas correlation between seedling PA and SLN varied substantially across years. Due to inadequate cold acclimation in 2022–2023 and heavy snow cover in 2023-2024, there was no significant correlation between FT levels and seedling PA during these periods. A significant negative correlation was observed between PA and SLN during the 2024–2025 season, indicating that more prostrate growth habits were associated with a reduction in leaf necrosis. These results indicate that architectural traits may contribute to FT only within certain environmental contexts. Thus, enhancing freezing tolerance should focus on direct survival tests in various environments, with secondary traits like SLN and PA considered as context-dependent factors.

## Introduction

Wheat (*Triticum aestivum* L.) is one of the world’s principal food crops, supplying approximately 20% of daily calories and 25% of dietary protein for about 35% of the global population (Faostat, 2024). Freezing injury is a major constraint to winter wheat production, particularly in China’s Huang-Huai-Hai Plain, where frequent freeze damage occurs. Recent climate trends have increased the frequency of extreme cold events, elevating the risk of large-scale freezing injury (Qian et al., 2014; Zhao et al., 2020; Zhang et al., 2024). To address this challenge, precise assessment of winter wheat freeze tolerance (FT) become essential for developing effective breeding strategies and producing management practices.

The geographic distribution of freeze-tolerant winter wheat is influenced by a complex interplay of climate, edaphic, and agronomic factors. In high-latitude regions with severe winters, such as northern China, distinct gradients of FT have evolved between winter and spring wheat types. This adaptation is quantifiably demonstrated by the 50% lethal temperature (LT_50_), a key metric for FT. In low-temperature chamber, the winter wheat cultivar Jing 411 exhibits robust hardiness with an LT_50_ of –21.2 °C, in contrast to the spring wheat Yanzhan 4110, which has a significantly higher LT_50_ of –14.7 °C. This nearly 7 °C difference in cold tolerance critically influences their suitable cultivation zones and demonstrates the role of local environmental selection pressures (Mu et al., 2015). While the FT of identified varieties demonstrates commendable reproducibility under controlled conditions, its application to field conditions presents significant challenges. This difficulty arises because, at the scale of field planting, assessing the FT of wheat varieties necessitates consideration of not only complex environmental factors but also economic costs.

Field-based classification of wheat freezing injury is commonly based on its seasonal timing and environmental co-factors. These primarily include a sudden early-winter temperature drop before full cold acclimation, prolonged mid-winter freezing that depletes carbohydrate reserves, freeze-drought events where frozen soil prevents water uptake, and alternating freeze–thaw cycles in early spring that lead to dehardening and damage to vulnerable young tissues (Zheng and Qi, 1984). This precise categorization is critical for developing tailored solutions, as the physiological basis of damage differs for each type, guiding both targeted breeding for specific stress tolerance and the implementation of season-specific management practices.

The traditional assessment of freezing injury in the field relies on manual visual scoring, assigning numerical levels based on visible signs of freezing injury in breeding programs. The mortality rate of shoots (MRS) is a widely utilized metric for variety approval in China (Liao et al., 2007). This approach requires cultivating wheat in cold, high-latitude regions and entails extensive manual examination to ascertain the tiller mortality. Although the relationships between MRS and extreme values and duration of low-temperature stress have been established in growth chamber (Mu et al., 2015; Cheng et al., 2025), it is difficult to simulate daily changing patterns in the field when freezing injury events occurred. Moreover, manual scoring is time-consuming and labor-intensive, especially for large field trials involving numerous samples.

Additional indicators, such as yield loss, severity of leaf necrosis (SLN), and photosystem II (PSII) photochemical efficiency, provide complementary data. Together, these metrics are instrumental in defining regional planting boundaries and assessing freeze-risk levels for winter wheat (Sun et al., 2013; Wu et al., 2024; Rapacz et al., 2015). Under low temperature stress, the leaves of wheat seedlings are folded, bent, and gradually necrosis and dried, which is consistent with freezing injured. SLN is frequently employed as an alternative to MRS in field and is extensively utilized to assess the FT of winter wheat during the tillering stage (Sun et al., 2013; Shammi et al., 2023; Sun et al., 2025). The SLN method is more straightforward than the MRS approach and is well-suited for machine learning applications in image recognition (Fu et al., 2021; Shammi et al., 2023; Sun et al., 2025). However, the relationship between the two methods, SLN and MRS, remains inadequately understood.

The plant architecture (PA) of wheat seedling, defined by traits such as tiller number, tiller angle, plant height, and leaf area, is an important factor in FT. Leaves, as the primary photosynthetic organs, directly influence cold hardiness through their structural and physiological traits affect the plant’s capacity to withstand cold stress. Furthermore, a hierarchical tolerance exists within the plant, with basal tillers exhibiting greater FT than their apical counterparts, likely due to their proximity to the soil which provides thermal insulation and protection (Xu et al., 2013). Prior to the onset of winter, PA of wheat seedlings may be both a result of the cold acclimation process and a functional adaptation that enhances the seedling’s ability to withstand freezing stress. FT coupled to winter growth habit was found to be associated with prostrate growth type in winter wheat (Canè et al., 2014) or cultivars with low vernalization requirements but high photoperiod response (Nishio et al., 1962). A commercial cultivar must undergo Distinctness, Uniformity, and Stability (DUS) testing; consequently, its erect/prostrate growth characteristics during the tillering stage are typically genetically stable and exhibit minimal susceptibility to environmental influences. The broad sense heritability of prostrate/erect growth habit in durum wheat was very high and ranged from 0.79 to 0.99, making possible analyses for single environment (Marone et al., 2020). A high broad-sense heritability (80%) of this trait was also reported in chickpea (Upadhyaya et al., 2017). The prostrate growth habit displayed prior to winter is an adaptive response proposed to suppress weed growth, reduce soil water evaporation, increase photosynthetic efficiency, and enable plants to benefit from warmer ground-level temperature and reduced wind exposure (Tan et al., 2008). However, it remains uncertain whether the FT of winter wheat varieties can be enhanced through the selection of appropriate PA at the tillering stage.

Increasing temperature variability under climate change has complicated the prediction and management of winter wheat freezing stress. In this study, we conducted a multi-season filed evaluation of 550 wheat varieties and advanced lines from the Huang-Huai-Hai winter wheat region of China. This study aims to elucidate the relationship between the two FT methods, SLN and MRS, as well as the association between FT and PA in wheat at tillering stage. Furthermore, the study seeks to identify superior germplasm and to inform the development of winter wheat cultivars with appropriate seedling PA and better FT.

## Materials and methods

### Plant materials

The study evaluated 550 winter wheat cultivars and advanced lines from China’s Northern and Huang-Huai winter wheat zones (**Supplementary Table S1**), where freezing damage frequently occurs.

### Field trials and phenotyping

Field trials were conducted over three consecutive growing seasons (2022–2023, 2023–2024, and 2024–2025) in Gaocheng, Hebei Province (114.76° E, 37.95° N). The experiment was arranged in a randomized complete block design (RCBD) with two blocks. Within each block, all materials were randomly assigned to experimental plots. Each plot consisted of one 3-m-long rows with 25 cm spacing between rows and 2 cm spacing between plants within a row.

**Figure 1:**
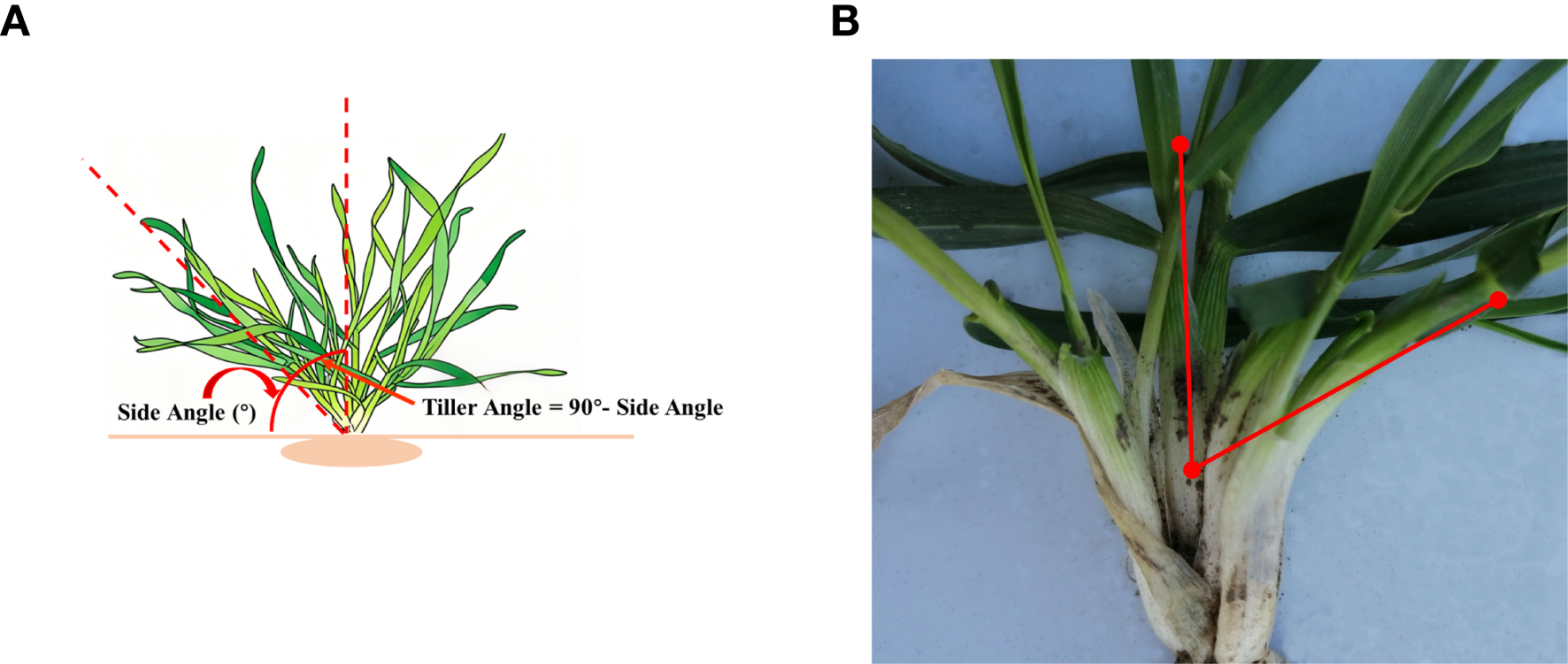
The tiller angle was measured as the angle between the outermost tillers and an imaginary vertical axis. **(A)** Schematic diagram of wheat tiller angle. The tiller angle was defined relative to two reference planes: the soil level (horizontal) and its normal (vertical). The angle between the outermost tiller and the vertical is the tiller angle; the complementary angle to the horizontal is the side angle. **(B)** Tiller angle values measured by crop angle meter (Zhejiang Top Cloud-agriculture Technology Co., Ltd., Hangzhou, Zhejiang Province). The nadir of the vertical red line is positioned at the soil level.

**Figure 2:**
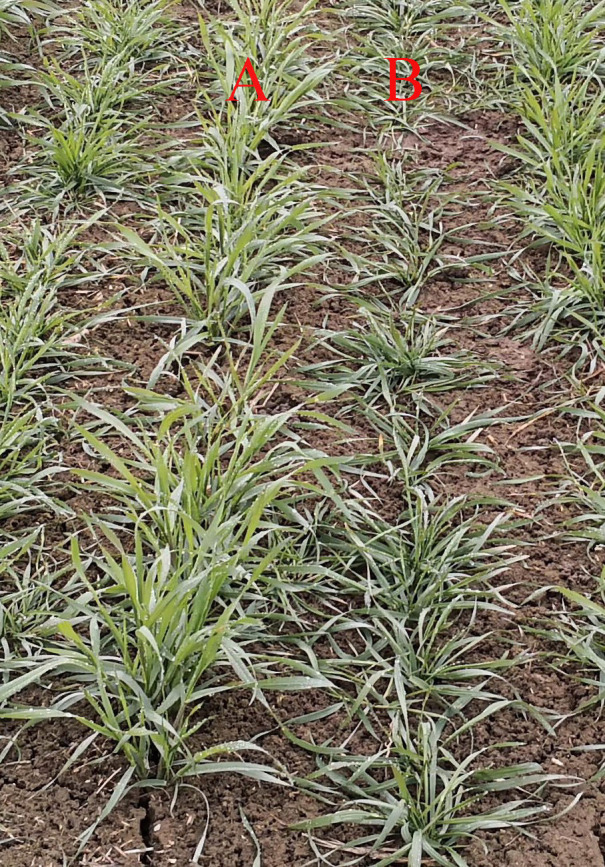
Examples of two genotypes with erect **(A)** and prostrate **(B)** growth habits at tillering stage. The photo was taken in early December 2024, during a period when the daily average temperature approached 0 °C.

During the 2024–2025 season, plant architecture (PA) was assessed in early December, when seedling growth ceased and mean daily temperature was near 0 °C. This timing corresponds to the maximum tillering stage, where tiller development is complete, ensuring consistent classification. The tiller angle, a key component of PA, was measured as the angle between the outermost fully expanded leaf of tiller and an imaginary vertical axis (**Figure 1A**) using a crop angle meter (Zhejiang Top Cloud-agriculture Technology Co., Ltd., Hangzhou, Zhejiang Province). Five randomly selected individuals were dug up one by one from each row and placed on the device platform for photography. Image recognition was then conducted, and subsequently, an angle value was generated (**Figure 1B**). The mean PA value from two replicates was used for final analysis. Based on these angle measurements, seedling PA was categorized into five distinct types according to the International Union for the Protection of New Varieties of Plants (UPOV, 2022) classification standards(https://www.upov.int/documents/d/upov/tg-documents-en-tg003.pdf) (**Supplementary Table S2**). Representative phenotypes for the erect and prostrate germplasm are shown in **Figure 2**.

**Figure 3:**
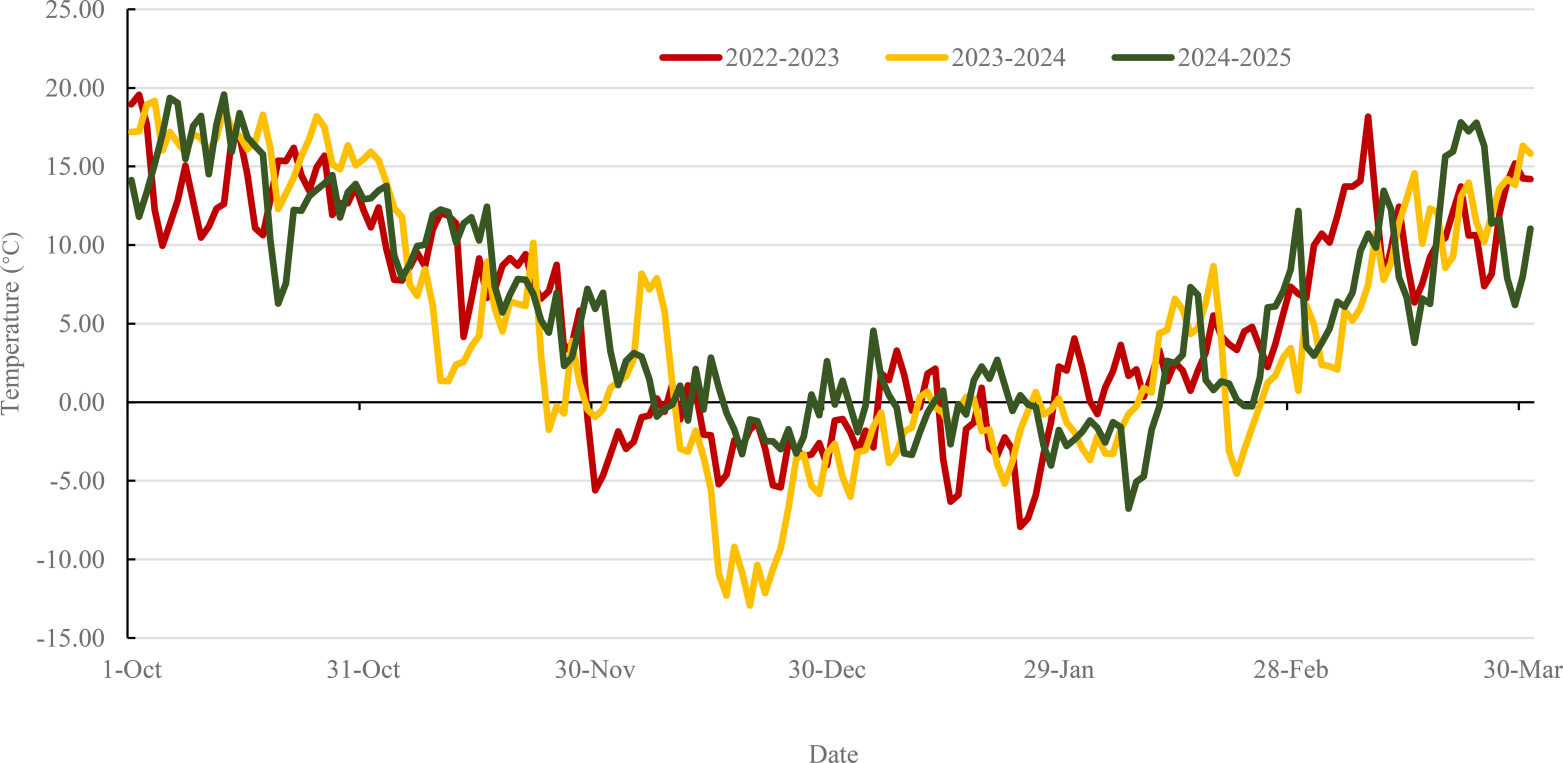
Ambient air average temperature dynamics during the wheat seedling stage across three growing seasons. The colors red, yellow, and green represent the periods from October 1st of the current year to March 31st of the subsequent year for 2022-2023, 2023-2024, and 2024-2025, respectively. In late November 2022, a sudden temperature drop occurred before seedling had completed cold acclimation. In mid-December 2023, heavy snow fell before the maximum temperature drop occurred.

**Figure 4:**
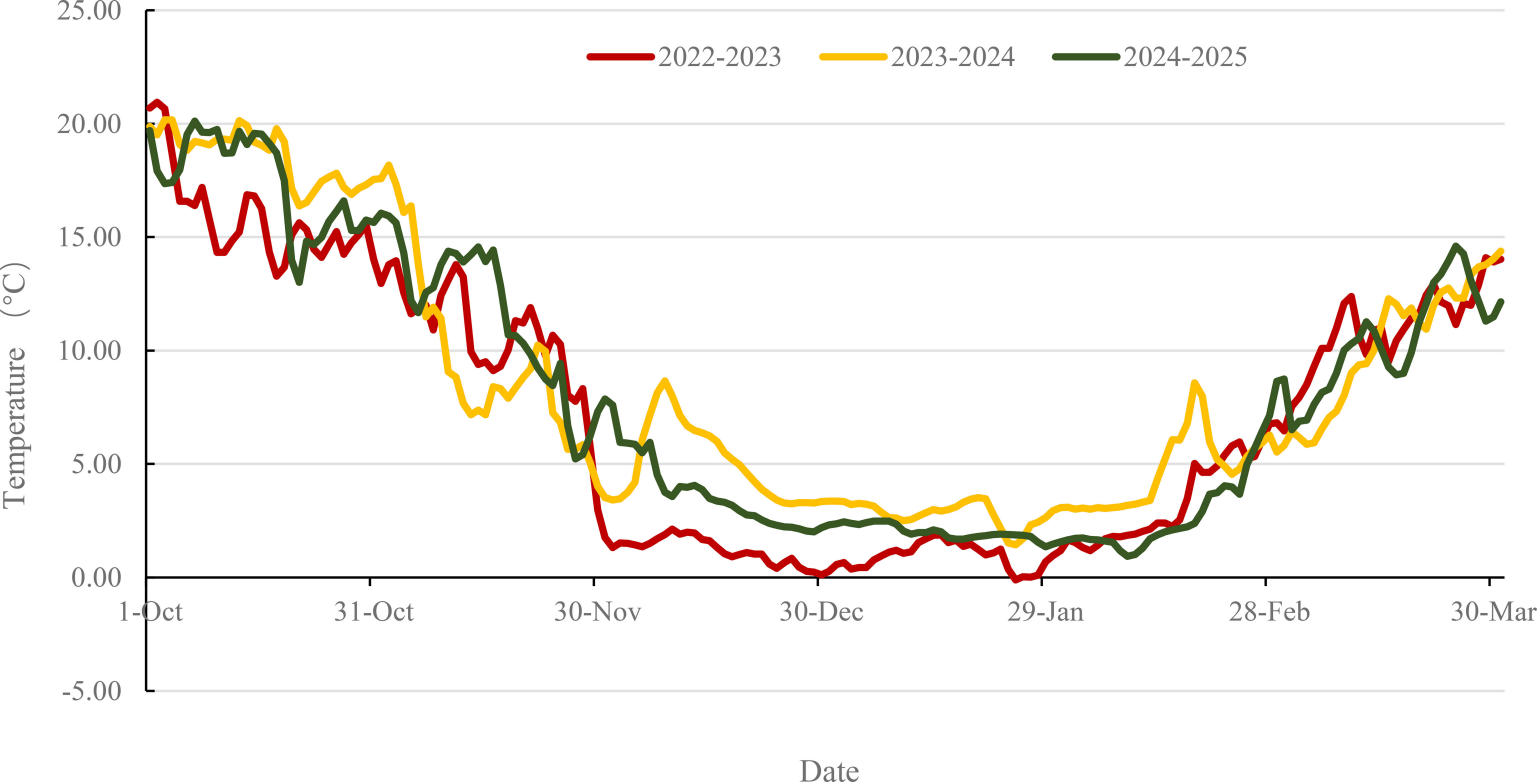
Soil average temperature (5 cm) dynamics during the wheat seedling stage across three growing seasons. The colors red, yellow, and green represent the periods from October 1st of the current year to March 31st of the subsequent year for 2022-2023, 2023-2024, and 2024-2025, respectively. Despite the significant decrease in air temperature recorded in December 2023 (**Figure 3**), the soil temperature remained relatively high (**Figure 4**). This phenomenon can likely be attributed to the insulating properties of snow cover, which mitigate the impact of rapid atmospheric fluctuations on subsurface layers.

Following winter dormancy, freeze tolerance (FT) was evaluated during the green-up period in mid-March, when mean daily temperatures reached approximately 6 °C. Meteorological data obtained from the weather station of the Institute of Cereal and Oil Crops, Hebei Academy of Agricultural and Forestry Sciences, which is situated within 2 km of the experimental plots(**Supplementary Table S3**). Fluctuations in ambient air temperature and soil temperature at a depth of 5 cm during the seedling stage across the three growing seasons are depicted in **Figures 3**, **4**, respectively. FT was assessed using two established metrics: severity of leaf necrosis (SLN) and mortality rate of shoots (MRS). The SLN method involves visual evaluation of leaf necrosis during early spring green-up when new heart leaves reach 1–2 cm in length according to Sun et al. (2013). The extent of above-ground necrosis was recorded and used to classify FT into five levels (**Supplementary Table S4**). For MRS, a 0.5 m section was excavated from each row. The total numbers of plants, shoots, and dead shoots were counted according to Liao et al. (2007). MRS was calculated for each replicate as the percentage of deceased shoots out of the total number of shoots. The mean MRS value from two replicates was used for final analysis and classified into five levels (**Supplementary Table S5**).

### Statistical analysis

Data management and basic descriptive statistics, including mean values, standard deviations, and percentage for PA, SLN, and MRS, were performed using Microsoft Excel 2019. All subsequent statistical analyses were conducted in SPSS (version 29.0, IBM Corp.). Pearson correlation analysis was used to examine relationships among PA, SLN, and MRS. A one-way analysis of variance (ANOVA) was employed to test for significant differences in SLN and MRS across the three growing seasons, with *post-hoc* comparisons conducted using Tukey’s HSD test where appropriate. Statistical significance was defined at α = 0.05, and all tests were two-tailed. Effect sizes are reported where applicable to complement significance testing.

## Results

### Distribution of seedling plant architecture from erect to prostrate at the tillering stage

**Figure 5:**
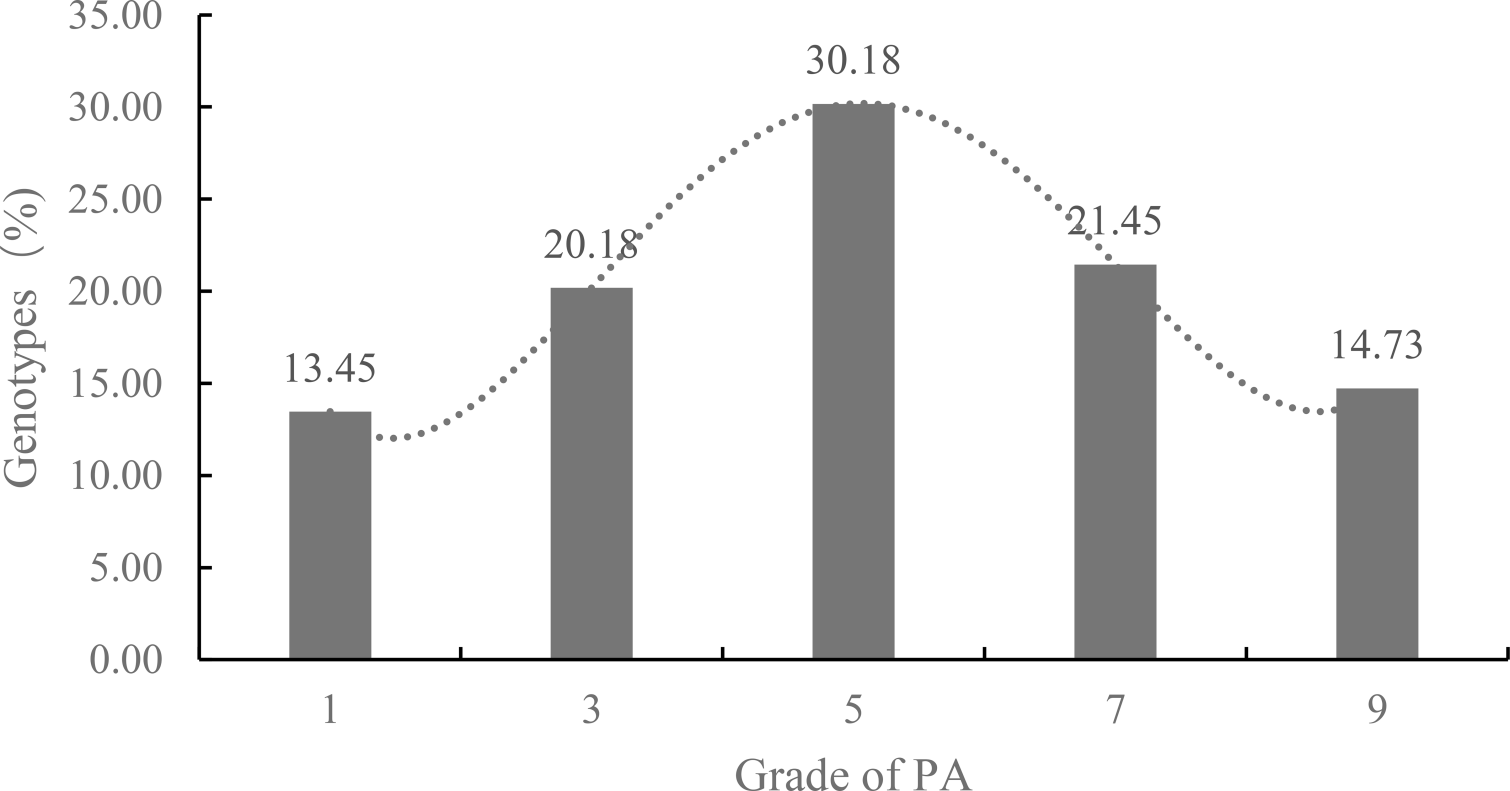
Percentage of seedling PA grades among 550 wheat germplasms at tillering stage. PA was categorized into five grades according the maximum angle between lateral tiller and vertical axis. 1: ≤ 20°, Erect; 3: 20.1°— 30°, Semi-erect; 5: 30.1°— 40°, Intermediate; 7: 40.1°— 50°, Semi-prostrate; 9: > 50.1°, Prostrate. Values on bars indicate the percentage of germplasms in each grade. The dotted line represents a fourth-order polynomial trendline generated by Excel, indicating that the distribution of PA across the population closely approximates a normal distribution.

PA was evaluated for 550 wheat accessions during the 2024–2025 growing season. The PA distribution across the population approximated a normal distribution (**Figure 5**). Among the five PA grades assessed, Grade 5 was most prevalent, comprising 30.18% (n = 166) of the accessions. The remaining grades accounted for 13.45% (Grade 1), 20.19% (Grade 3), 21.45% (Grade 7), and 14.73% (Grade 9), respectively.

### Evaluation of freeze tolerance by severity of leaf necrosis and mortality rate of shoots

#### Distribution of freeze tolerance levels across growing seasons

**Figure 6:**
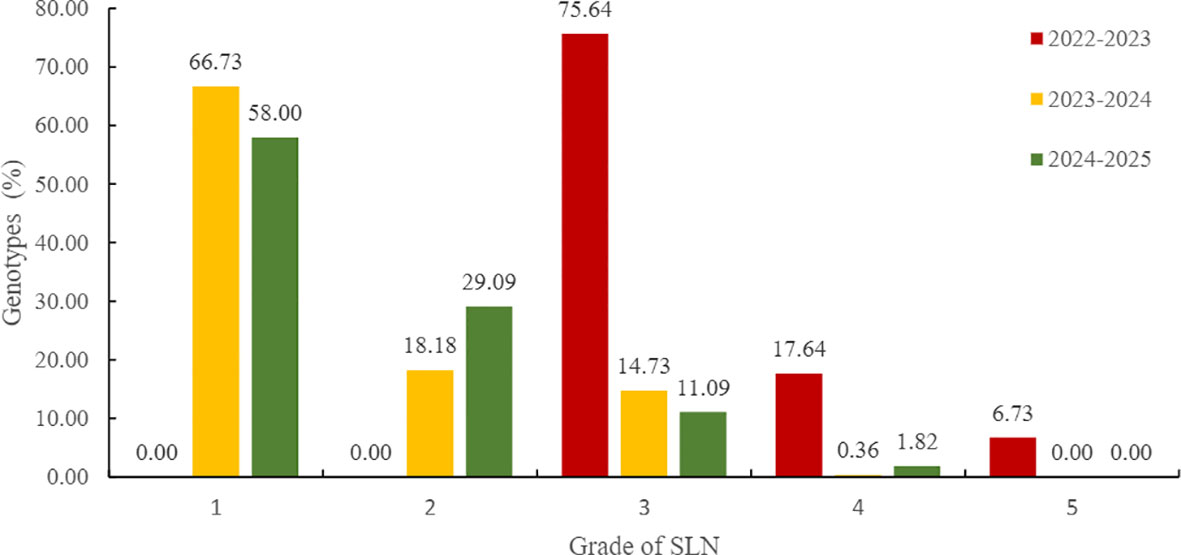
The SLN-based freeze tolerance of 550 wheat germplasms across three planting years. 1–5 scale of freeze tolerance is based on severity of leaf necrosis (SLN). Values on bars indicate the percentage of germplasms in each grade. The colors red, yellow, and green represent the growing seasons of 2022-2023, 2023-2024, and 2024-2025, respectively.

FT was evaluated using both SLN and MRS methods across three growing seasons. Both assessment systems classified FT into five ordinal grades (1-5), with Grade 1 representing highest tolerance. The distribution of FT grades varied between assessment methods and across years. SLN-based FT evaluation revealed a shift in tolerance distribution. In 2022-2023, most accessions (75.64%) clustered at Grade 3. Grades 4 and 5 represented 17.64% and 6.73%, respectively. In subsequent seasons, Grade 1 accessions increased to 66.73% (2023-2024) and 58.00% (2024-2025), respectively (**Figure 6**).

**Figure 7:**
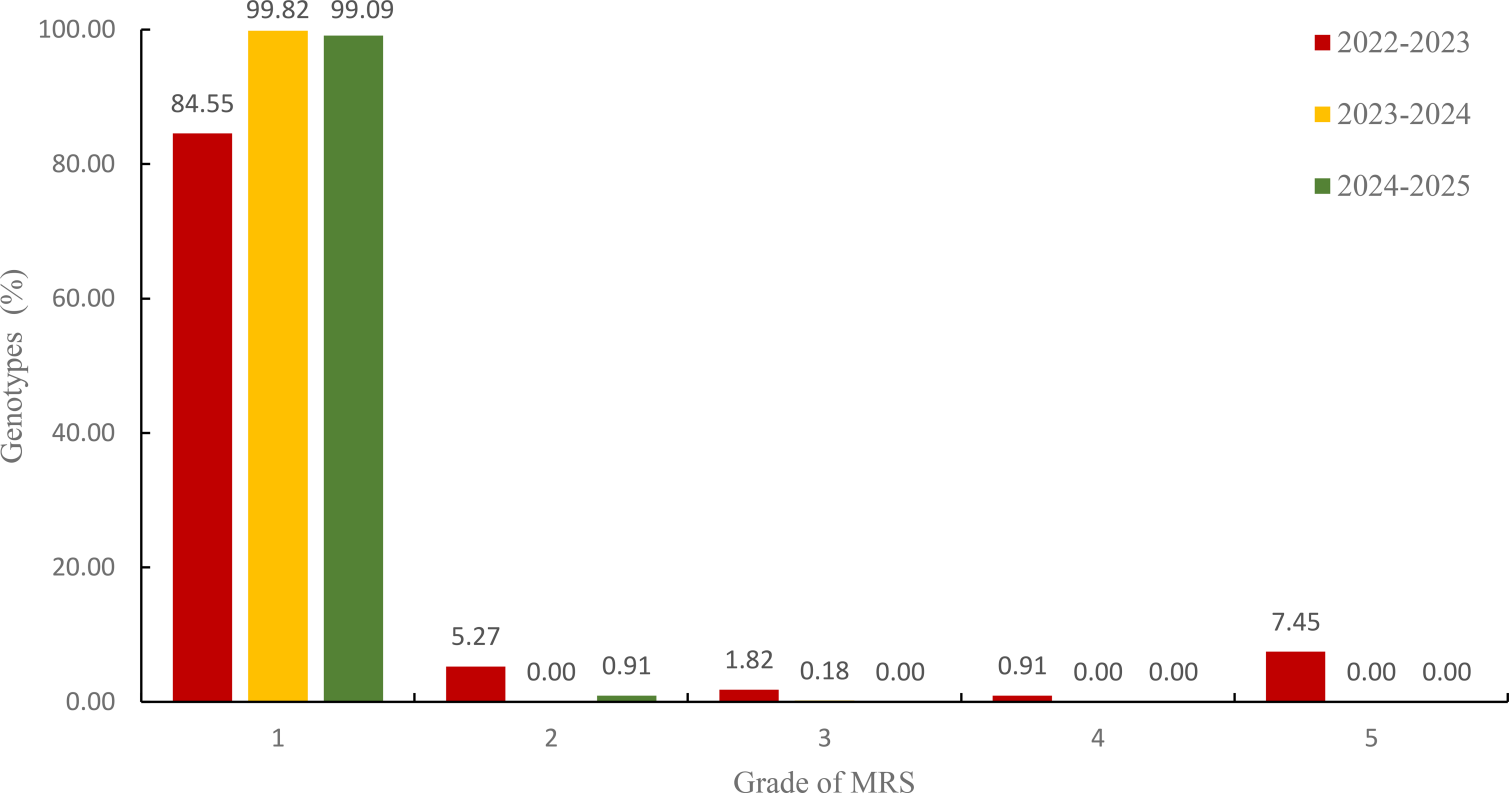
The MRS-based freeze tolerance of 550 wheat germplasms across three planting years. 1–5 scale of freeze tolerance is based on mortality rate of shoots (MRS). Values on bars indicate the percentage of germplasms in each grade. The colors red, yellow, and green represent the growing seasons of 2022-2023, 2023-2024, and 2024-2025, respectively.

In contrast, MRS assessment demonstrated consistently higher tolerance ratings across all seasons. The majority of accessions (84.55-99.82%) achieved Grade 1 classification, with minimal representation in lower tolerance grades (**Figure 7**). This discrepancy between two assessment methods suggests differential sensitivity to freezing stress indicators.

#### Correlation between severity of leaf necrosis and mortality rate of shoots

The correlations between SLN and MRS were positive and statistically significant, with correlation coefficients of 0.343 in 2022-2023, 0.510 in 2023-2024, and 0.760 in 2024-2025 (*P* < 0.01). This confirms that both methods are valid indicators of FT under the same condition.

Many accessions that exhibited severe leaf damage (high SLN) in 2022–2023 demonstrated high survival rates (low MRS) in three consecutive growing seasons. Meteorological data indicate that ambient air temperatures are consistently lower than soil temperatures (**Figures 3**, **4**). Consequently, leaves are likely to experience freezing stress compared to the crown of tillers, which are situated 2–3 cm below the soil surface, thereby increasing their susceptibility to freezing and potential mortality.

#### Interannual variation in freeze tolerance

Analysis of variance confirmed significant differences in both MRS and SLN values across the three growing seasons (*P* < 0.01). Mean MRS values decreased progressively from 6.39% in 2022–2023 to 0.36% in 2023–2024 and 0.19% in 2024-2025 (**Table 1**). Similarly, mean SLN values declined from 3.31 to 1.49 and 1.56 across the same periods (**Table 1**). While both metrics showed consistent interannual patterns in FT, SLN demonstrated greater sensitivity to year-to-year environmental variation, reflecting its responsiveness to specific stress events during cold acclimation period.

**Table 1:** Variance analysis of MRS (%) and SLN across three planting years.

Evaluation method of FT	Annual	Sample size	Average	SD	*F*	*p*
MRS (%)	2022-2023	550	6.39	15.04	89.529	0.000**
	2023-2024	550	0.36	1.37		
	2024-2025	550	0.19	1.39		
	Sum	1650	2.31	9.21		
Grade of SLN	2022-2023	550	3.31	0.59	1178.883	0.000**
	2023-2024	550	1.49	0.75		
	2024-2025	550	1.56	0.77		
	Sum	1650	2.12	1.10		

**The difference is extremely significant at *P* < 0.01. MRS, Mortality rate of shoots. SLN, Severity of leaf necrosis.

### Relationship between freeze tolerance and seedling plant architecture

The correlation between seedling PA and FT varied substantially across years. In both 2022–2023 and 2023-2024, no significant correlation was detected between PA and SLN. The lack of relationship in 2022–2023 coincided with a sudden temperature drop prior to seedling cold acclimation, while in 2023-2024, protective snow cover likely mitigated freezing damage(**Supplementary Tables S3–1, S3-2**). In the 2024–2025 season, an extremely significant negative correlation emerged between PA and SLN (*P* < 0.01; **Table 2**), indicating that more prostrate growth habits were associated with reduced leaf necrosis. The variations across different years indicate that winter temperatures are not the sole predominant factor affecting the PA-SLN relationship. MRS showed no significant correlation with PA across all three growing seasons, suggesting that ultimate shoot survival remains independent of seedling architecture.

**Table 2:** Correlation analysis between freeze tolerance and plant architecture of wheat seedling.

Level of FT	Grade of PA		
	2022-2023	2023-2024	2024-2025
SLN	0.033	0.027	-0.202^**^
MRS	-0.015	-0.07	-0.034

**The correlation is highly significance at *P* < 0.01. MRS, Mortality rate of shoots. SLN, Severity of leaf necrosis.

Over the three growing seasons, several freeze-tolerant varieties were identified across the diverse panel of 550 accessions (as detailed in **Supplementary Table S1**). Notably, these tolerant varieties were present across all PA categories, including erect (e.g., Zhongmai 7158), semi-erect (e.g., Nongda 1980), intermediate (e.g., Jimai 479), semi-prostrate (e.g., Xingmai 29), and prostrate (e.g., Henong 405) types. This finding indicates that genetic freezing tolerance can be achieved independently of specific PA, highlighting the complexity of this trait and the likelihood of multiple, compensatory physiological and molecular mechanisms underlying survival. Consequently, these results imply that breeding for FT should not be restricted to selecting a single, idealized PA. Instead, efforts should focus on integrating tolerance genes within a range of suitable, locally adapted plant architectures.

## Discussion

FT is a vital agronomic trait in winter wheat, fundamentally determining overwintering survival, subsequent growth, and final yield. This is particularly crucial in the North China Plain, where increasing frequency of extreme cold events heightens production risks (Wu et al., 2024). Consequently, cultivating wheat varieties with robust FT provides the most effective defense against frost damage. Precise evaluation of varietal FT enables more accurate determination of cultivation boundaries and provides essential guidance for breeding programs. Additionally, seedling PA, particularly tiller angle, constitutes an important agronomic trait with demonstrated effects on both ecological adaptation and yield formation (Marone et al., 2020), suggesting its potential interaction with freezing stress response.

### Intermediate seedling growth habit may contribute to freeze tolerance and high-yielding winter wheat

The seedling PA of the 550 winter wheat cultivars and advanced lines was predominantly characterized as intermediate to semi-prostrate. This growth habit appears to balance ecological adaptation with photosynthetic efficiency. The lower canopy profile reduces exposure to chilling winds, while maintaining adequate leaf area for light capture. Such an architectural balance likely supports both winter survival and biomass accumulation during early growth stages. Research in durum wheat has identified prostrate juvenile growth as a valuable trait for enhancing weed competitiveness and water conservation through improved ground coverage (Marone et al., 2020). Wheat leaves retained a complete photosynthetic apparatus, induced sustained nonphotochemical quenching (NPQ) during the cold period in winter, and relaxed NPQ rapidly during the warm period. The photosynthetic apparatus of wheat switched quickly between photosynthetic carbon assimilation and the photoprotective state during the winter (Li et al., 2025). Thus, selection for intermediate seedling PA represents a promising strategy for developing winter wheat varieties with enhanced FT and climate resilience without compromising yield potential.

### Prostrate growth habits enhance seedling freeze tolerance

A significant negative correlation between SLN and PA grade emerged during the 2024–2025 growing season, revealing that more prostrate seedling sustained less leaf necrosis. This finding aligns with observations in durum wheat, where prostrate growth habits reduced frost exposure (Marone et al., 2020). The genetic regulation of prostrate growth involves loci linked to vernalization and frost resistance genes on chromosome 5A (Roberts, 1990), resulting in compact plants characterized by increased tillering, narrower leaves, and reduced elongation (Li et al., 2016). Evidence shows that there are differences in the accumulation of cold-resistant substances between erect and prostrate wheat seedlings. Transcriptomics and nontargeted metabolomics analyses revealed that lignin biosynthesis, ABC transporters, sucrose and glycerophospholipid metabolism were key pathways underlying the differentiation of erect and prostrate growth habits in the wheat overwintering period (Cheng et al., 2025).

The morphological adaptation of a prostrate growth habit is frequently part of an evolutionary suite of traits in cold-adapted plants, working together to reduce physical exposure, protect meristems, and support the physiological and biochemical processes involved in cold acclimation.

### Cold acclimation and snow cover significantly impact the freeze tolerance of winter wheat

The magnitudes of FT among the 550 germplasms varied significantly across the three growing seasons, demonstrating the strong influence of climatic conditions on winter survival. Global climate change cause longer and warmer autumns, thus negatively affecting the quality of cold acclimation and reducing the FT of winter wheat (Rapacz et al., 2017). The development of FT depends critically on successful cold acclimation, a process increasingly disrupted by climate change as prolonged autumn warmth delays hardening, ultimately reducing cold hardiness. This effect was evident in late November 2022, when a sharp temperature drop occurred before acclimation was complete, resulting in significantly higher SLN and MRS values compared to subsequent seasons. Recent research corroborates that extended exposure to mild autumn temperatures, while potentially boosting shoot biomass, can concurrently diminish freezing resistance (Vaitkevičiūtė et al., 2022).

Snow cover also substantially moderated frost injury, as demonstrated in mid-December 2023 when continuous snowfall insulated the soil and minimized freeze-thaw cycling. The protective role of snow exhibits spatial dependence, with even minor variations in snow depth causing substantial differences in survival rates within single fields (Hayhoe and Andrews, 1999). For instance, winter wheat survival at the Motala site in Sweden improved significantly with snow depth increases from 0 to 5 cm (Bergjord-Olsen et al., 2018). These results collectively highlight that both temperature regimes during acclimation and snow cover dynamics are key determinants of field-based FT expression.

### The MRS method is a reliable approach for evaluating genetic freezing tolerance

The shoot apical meristem (SAM), situated near the soil surface within unelongated internodes, benefits from soil insulation and accumulate protective carbohydrates. This study confirmed that leaves are more freezing-sensitive than tillers, with intact tillers capable of regenerating new shoots even following complete leaf necrosis. The critical role of the crown region (2–3 cm below soil surface) in frost resistance is well established (Bao et al., 2021), with winter-hardy cultivars like ‘Nongda 211’ achieving LT_50_ temperatures as low as -21.2 °C (Wu et al., 2024). These results collectively demonstrate how soil microclimate and growth habits interact to enhance overwintering survival by protecting crown meristems.

During the winter season, atmospheric temperatures generally decrease to levels lower than those of the soil, rendering the leaves of the aerial plant parts more vulnerable to freeze damage compared to the subterranean SAM. In this study, MRS assessment demonstrated consistently higher tolerance ratings. SLN is more sensitive to cold weather than MRS, and it can further distinguish the FT of different varieties. Other alternate methods, such as LT50 assays, controlled freeze tests, electrolyte leakage, OJIP, NDVI, could improve discrimination on FT (Mu et al., 2015; Grabelnych et al., 2014; Rapacz et al., 2015; Shammi et al., 2023; Sun et al., 2025). While MRS serves as an unambiguous endpoint for plant survival, its correlation with SLN was inconsistent, suggesting tissue-level damage does not always predict whole-plant fate. Thus, the MRS provides a more reliable evaluation of genetic freezing tolerance compared to other methods.

## Conclusion

This three-year field evaluation of 550 wheat varieties and advanced lines from China’s Huang-Huai-Hai region revealed that seedling PA follows an approximately normal distribution. Although FT levels showed significant interannual variation, strong positive correlations between SLN and MRS assessments within same seasons confirmed the reliability of both evaluation methods. Due to the environmental differences between where the leaves and shoots are located, the SLN method is more sensitive to cold weather than the MRS method. The significant negative correlation between PA and SLN observed in 2024–2025 indicates that architectural traits may contribute to FT only within certain environmental contexts, rather than being a universally stable predictor. Therefore, breeding for improved freezing tolerance should rely on direct survival assays (MRS) across multiple environments; while considering secondary traits like SLN and PA as context-dependent contributors whose utility requires further mechanistic and multi-environment investigation. These results provide a foundation for developing winter wheat cultivars with optimized seedling architecture and enhanced freezing tolerance through targeted breeding strategies.

## Data Availability

The original contributions presented in the study are included in the article/**Supplementary Material**. Further inquiries can be directed to the corresponding authors.
